# Environmental sanitation satisfaction and associated factors among residents from National Sanitary Cities

**DOI:** 10.3389/fpubh.2026.1817824

**Published:** 2026-07-08

**Authors:** Manhui Zhang, Jiaxin Dong, Xian Xia, Jinfang Sun, Hongyan Yao, Jianjun Liu, Zhigang Wang, Qiqi Wang

**Affiliations:** 1 Department of Disease Control and Prevention, The Seventh Medical Center of Chinese PLA General Hospital, Beijing, China; 2 Beijing Center for Disease Prevention and Control, Beijing, China; 3 Office of Epidemiology (Technical Guidance Office for Patriotic Health Work), Chinese Center for Disease Control and Prevention (Chinese Academy of Preventive Medicine), Beijing, China; 4 Office of Education and Training (Graduate School), Chinese Center for Disease Control and Prevention, Beijing, China; 5 Chinese Center for Disease Control and Prevention (Chinese Academy of Preventive Medicine), Beijing, China

**Keywords:** associated factors, environmental sanitation, National Sanitary City, residents' satisfaction, survey research

## Abstract

**Objective:**

Residents are not only beneficiaries of National Sanitary City development but also participants who judge whether sanitation improvements translate into tangible daily benefits. This study assessed residents' subjective satisfaction with urban environmental sanitation and its associated factors to identify weaknesses relevant to people-centered sanitation governance.

**Methods:**

Between November 2021 and April 2022, a multistage sampling method was employed to evaluate the environmental sanitation satisfaction of 17,865 residents in National Sanitary Cities and non-National Sanitary Cities within four provincial-level administrative regions using a standardized structured questionnaire.

**Results:**

To meet the evaluation requirements, we selected 80 and 90% of the total scores as the criteria for judging satisfaction. At the 80% satisfaction cut-off point, 22.038% of residents expressed satisfaction with environmental sanitation, with residents in National Sanitary Cities showing a higher level of satisfaction (23.375%). Environmental sanitation satisfaction was associated with gender, educational level, age, occupation, living area, residence duration, and health literacy (*P* < 0.05). Residents in National Sanitary Cities also reported higher satisfaction levels at the 90% cut-off point (5.551 vs. 3.896%).

**Conclusion:**

The findings suggest that National Sanitary City status was more clearly related to high-level environmental sanitation satisfaction. Overall satisfaction remained low, and residents' perceptions were also shaped by demographic, socioeconomic, living-area, and regional contextual factors. Routine attention to residents' subjective perceptions can complement objective sanitation indicators and help refine people-centered policy actions.

## 1 Introduction

Urban environmental sanitation is a complex system (1). Poor sanitation contributes to the spread of public health-threatening diseases, posing significant risks to urban residents. The goal of improving urban environmental sanitation is to mitigate these risks by addressing the underlying factors that can lead to health issues (2). The livability of an area depends on its characteristics, geographical features, and the behaviors of its residents (3). In this context, human-induced environmental risks such as air pollution (4), water pollution (5), noise pollution (6), and solid waste management (7) have profound impacts on health. Thus, enhancing environmental sanitation is vital for the sustainable development and construction of urban areas.

The concept of satisfaction assessment originated in management, serving as a measure to evaluate the alignment between expectations and reality. Since the 1950s, satisfaction research has expanded into various fields, including healthcare and public health, where it is now employed to gauge the effectiveness of services (8). Recently, the focus on residents' satisfaction with their living environment has intensified. Research has explored satisfaction with urban parks (9, 10), greenways (11), and other residential amenities (12), investigating influencing factors (13, 14) and developing and assessing evaluation models (15).

Studies have increasingly treated residents as users and evaluators of urban environments, emphasizing that subjective perceptions reveal whether formal improvements are experienced as meaningful gains in daily life (16–18). Satisfaction is therefore a relevant outcome for environmental sanitation governance: objective infrastructure and administrative standards indicate whether a city has met technical requirements, while residents' satisfaction captures perceived improvement, lived convenience, and everyday wellbeing. Recent research on China's Sanitary City Initiative has also used residents' satisfaction to evaluate the built environment and highlighted the value of considering city-level context alongside individual characteristics (19).

The National Sanitary Cities initiative, a pivotal public policy in China, integrates the concept of healthy urban development with the country's core conditions, creating a unique system. This initiative acts as a comprehensive gauge of a region's development and civilizational standards, and it is essential for enhancing living conditions and the overall quality of life. Since the launch of the National Sanitary Cities initiative, significant progress has been achieved in building urban environmental sanitation infrastructure, optimizing sanitation governance, upgrading urban environmental appearances, boosting economic development, and raising residents' public health awareness. As of December 2020, a total of 462 National Sanitary Cities and 3,751 National Sanitary Counties have been designated, covering all 31 provinces, autonomous regions, and municipalities directly under the Central Government, as well as the Xinjiang Production and Construction Corps (20). Earlier studies highlighted the crucial role of government focus on residents' subjective experiences of environmental sanitation and the development of supportive health environments during urban construction for effective urban development (21).

The policy evaluated in this study should be distinguished from the broader WHO Healthy Cities framework. The WHO approach emphasizes health-supportive urban environments and governance for health, whereas China's National Sanitary City initiative is a China-specific designation and review program administered through patriotic health governance and assessed against national standards for sanitation, health promotion, key-place hygiene, ecological environments, public health facilities, disease prevention, and public participation (22, 23). These frameworks are related in their concern for health-supportive cities, but they are not identical policy instruments.

This distinction strengthens the policy rationale for examining residents' perceptions. National Sanitary City construction is implemented through government leadership, interdepartmental collaboration, and broad social participation. A people-centered city is built by and for its residents; accordingly, residents should not be treated as passive beneficiaries of sanitation upgrades. Their satisfaction is a crucial dimension of policy evaluation because it indicates whether visible infrastructure, management routines, and formal assessment results have been converted into perceived gains in daily life.

This study compares residents' environmental sanitation satisfaction in National Sanitary Cities and non-National Sanitary Cities and examines associated individual- and region-level factors. By foregrounding residents' subjective experiences alongside the policy context of National Sanitary City construction, the study aims to identify gaps that may be missed by objective indicators alone and to inform more targeted, people-centered sanitation governance.

## 2 Methods

### 2.1 Data sources and study sample

The survey was conducted between November 2021 and April 2022. This study examined National Sanitary Cities and non-National Sanitary Cities using a multistage sampling design in Hainan, Guizhou, Guangxi, and Sichuan. These four provincial-level regions were selected to reflect the research focus on areas facing ongoing challenges in National Sanitary City construction while balancing contextual heterogeneity, field feasibility, budget, and logistics. Inclusion criteria for residents were: residency in the survey area for 6 months or more; age 18 years or older; clear cognitive and comprehension abilities; and willingness to participate in the questionnaire survey.

**Figure 1:**
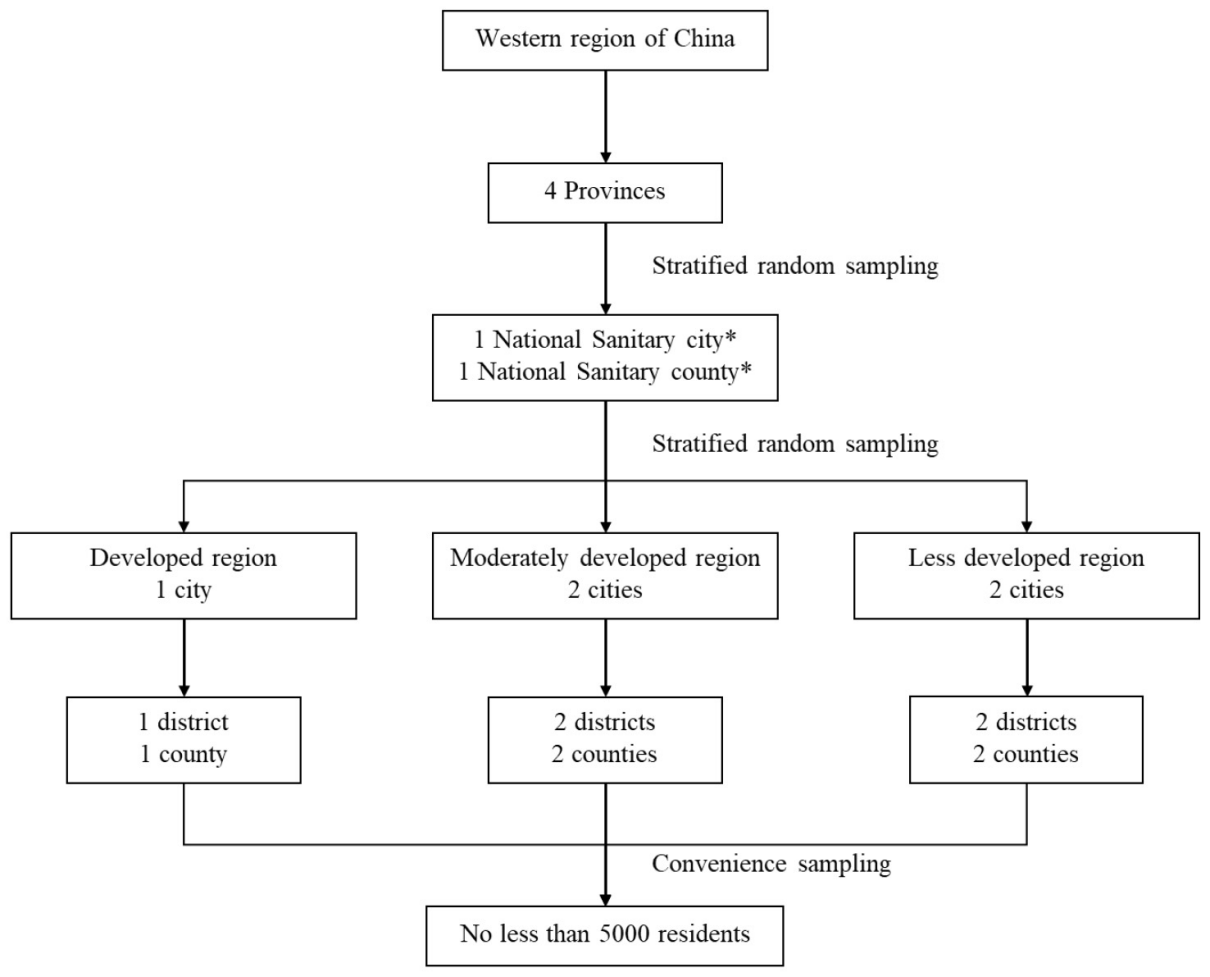
Sampling scheme. *In this stage, cities designated as National Sanitary Cities or National Sanitary Counties were selected, and subsequently, one district or one county is correspondingly reduced in the subsequent sampling.

The multi-stage stratified sampling approach was employed (Figure 1).

In the first stage of sampling, the four provincial-level regions were selected according to the research objectives, the characteristics of each region, budget constraints, and logistical limitations.

In the second phase, based on the national per capita Gross Domestic Product (GDP) in 2019, the cities within the selected provinces were ranked according to their GDP levels and divided into three tiers: developed areas, moderately developed areas, and underdeveloped areas, by equal quantile division. Since our study focuses more on the environmental satisfaction in underdeveloped areas, one city was randomly selected from the developed areas, and two cities were randomly selected from both the moderately developed and underdeveloped areas.

In the third phase, to ensure that each selected district and county in the provinces included one National Sanitary City and one National Sanitary County, we first randomly selected one National Sanitary City and one National Sanitary County in each selected city within the chosen provinces. In the selected cities that did not have a district/county drawn, we drew districts/counties based on demand, ensuring that each selected city has one district and one county included in the study. The provinces selected for the study include Hainan, Guizhou, the Guangxi Zhuang Autonomous Region, and Sichuan, with a total of 40 districts/counties chosen.

Then, stratified sampling was conducted based on gender and age to ensure that the number of participants in each stratum closely matches the age and gender structure of the local population. The sample size calculation was conducted using the following formula:


$$\begin{array}{l} N=\frac{u_{\frac{\alpha }{2}}^{2}\times \pi \times \left(1-\pi \right)}{\delta^{2}}\times deff \end{array}$$

The meanings and values of the parameters are as follows: the confidence level is taken at 95% (two-tailed), with the corresponding *u* = 1.96; the probability π is taken as the resident satisfaction rate of 20%; the design effect (*Deff* ) was set to 3; the allowable error δ = 2%. Based on the values of these parameters, the calculated sample size is 4,609 people. Considering a non-response rate of 10%, the total required sample size is approximately 5,000 people.

According to the sampling population data from various districts and counties in 2019, the gender and age distribution of the sampled population is consistent with the total population.

National Sanitary Cities and National Sanitary Counties are designated according to national administrative divisions, with each receiving their designation separately. The scope of National Sanitary Cities includes prefecture-level administrative bodies and county-level cities (“district” in Figure 1); National Sanitary Counties include counties (“county” in Figure 1). Since National Sanitary Cities are reviewed uniformly by the national standards, while National Sanitary Counties are reviewed individually by each province, in this study, we only used data at the city level (areas designated as National Sanitary Cities or corresponding to “district”). The content of the questionnaire survey does not involve sensitive information, and informed consent has been obtained from the subjects of the survey.

### 2.2 Study variables

The survey was conducted using the “Resident Environmental Sanitation Satisfaction Survey.” This instrument gathered basic respondent demographics, general information about the survey area, and assessed satisfaction levels concerning environmental sanitation, including urban aesthetics, sanitation management, water sanitation, and sanitation in key public places. It also identified issues in environmental sanitation as perceived by the respondents. Survey responses were evaluated using a Likert 5-point scale: 1 for very dissatisfied, 2 for dissatisfied, 3 for neutral, 4 for satisfied, and 5 for very satisfied. For detailed information about the questionnaire and its reliability and validity, please refer to the published results of the research team (24, 25).

Residents' satisfaction scores were represented by the summed questionnaire score. Because the questionnaire used a 5-point Likert scale and a score of 4 on each item indicates being satisfied, 80% of the maximum total score corresponds to an average item score of 4 and was selected as the primary policy-relevant threshold for basic satisfaction. The 90% threshold was used as a stricter benchmark for high-level satisfaction, reflecting a level between generally satisfied and very satisfied across questionnaire items. This dual-threshold approach allows the analysis to distinguish factors associated with reaching basic satisfaction from those associated with achieving a higher satisfaction standard. To reduce information loss from dichotomization, the original continuous total score was also analyzed as a robustness check.

Data collection included basic demographic information (age, gender, educational level, occupation, and living area) obtained through questionnaires and health metrics (health literacy rate, average years of education, and infant mortality rate) captured through the Patriotic Health Information Management System. The system supports reporting and management for patriotic health work through records on organization, annual regional information, designations, assessment, evaluation, expert resources, and tobacco-control activities. For cities lacking complete data, we extracted supplementary data from the China Health Statistical Yearbooks.

### 2.3 Quality control

Before the commencement of the survey, investigators underwent rigorous training to standardize the completion of the questionnaire and were only officially deployed after passing the assessment. Investigators introduced the purpose of the survey to the subjects and obtained their consent before proceeding. Subjects filled out the questionnaires on their own, while the older adults and residents with lower levels of education completed the questionnaires under the guidance of the investigators. A quality check system was established and implemented to assess and evaluate the quality of the survey according to the survey locations and populations.

Data were entered and verified using EpiData 3.1 software (The EpiData Association, Odense, Denmark) in a double-entry mode. Questionnaires with severe data missing were excluded to ensure the rigor and accuracy of the data.

### 2.4 Statistical analysis

Quantitative data were analyzed using the Mann–Whitney *U* test and presented as median (P25, P75), while categorical data were analyzed using the chi-square test and expressed as *n* (%). Descriptive analysis was used to describe the study sample. The original continuous satisfaction score was analyzed using OLS regression as a robustness analysis, and the two threshold-defined binary outcomes were analyzed using multivariable logistic regression. Multicollinearity was assessed using variance inflation factors (VIFs). All statistical tests were two-tailed, and *P* < 0.05 was considered statistically significant. SPSS version 22.0 (IBM Corporation, Armonk, NY, USA) facilitated the statistical analysis.

## 3 Results

This study employed a standardized structured questionnaire to assess environmental sanitation satisfaction among 17,865 residents from four provincial-level administrative regions. Of the survey participants, 7,034 were male (39.373%), 11,549 had at least a junior college or bachelor's degree (64.646%), 9,101 were under 40 years old (50.943%), and 9,542 had resided in the survey area for over 10 years (53.412%). Univariate analysis indicated significant group differences at the 80% cut-off point for gender, educational level, age, occupation, living area, living time, regional economic situation, health literacy rate, average years of education, and infant mortality rate (*P* < 0.001); age and living time were not significant at the 90% cut-off point. The survey encompassed 11 cities, including nine National Sanitary Cities. At the 80% cut-off point, 2,754 residents from National Sanitary Cities reported satisfaction (23.375%), compared with 1,183 residents from non-National Sanitary Cities (19.448%). At the 90% cut-off point, the corresponding proportions were 5.551 and 3.896% (Table 1).

**Table 1:** Basic information on residents' environmental sanitation satisfaction in National Sanitary Cities and non-National Sanitary Cities (*n* = 17,865).

Variable	Total	80% score as cut-off point			90% score as cut-off point		
		Satisfied	Unsatisfied	*P* value	Satisfied	Unsatisfied	*P* value
Gender, *n* (%)				**<0.001**			**<0.001**
Male	7,034 (39.373)	1,879 (26.713)	5,155 (73.287)		451 (6.412)	6,583 (93.588)	
Female	10,831 (60.627)	2,058 (19.001)	8,773 (80.999)		440 (4.062)	10,391 (95.938)	
Educational level, *n* (%)				**<0.001**			**<0.001**
Junior high school and below	2,843 (15.914)	521 (18.326)	2,322 (81.674)		85 (2.990)	2,758 (97.010)	
Technical secondary school/senior high school/technical school	3,473 (19.440)	749 (21.566)	2,724 (78.434)		149 (4.290)	3,324 (95.710)	
Junior college/bachelor degrees and above	11,549 (64.646)	2,667 (23.093)	8,882 (76.907)		657 (5.689)	10,892 (94.311)	
Age, *n* (%)				**<0.001**			0.101
18–40	9,101 (50.943)	1,894 (20.811)	7,207 (79.189)		438 (4.813)	8,663 (95.187)	
41–60	6,962 (38.970)	1,634 (23.470)	5,328 (76.530)		375 (5.386)	6,587 (94.614)	
≥60	1,802 (10.087)	409 (22.697)	1,393 (77.303)		78 (4.329)	1,724 (95.671)	
Occupation, *n* (%)				**<0.001**			**<0.001**
Managers of government agencies, enterprises and institutions	3,849 (21.545)	1,134 (29.462)	2,715 (70.538)		324 (8.418)	3,525 (91.582)	
Professional technicians	5,314 (29.745)	1,029 (19.364)	4,285 (80.636)		231 (4.347)	5,083 (95.653)	
Retired	1,374 (7.691)	333 (24.236)	1,041 (75.764)		55 (4.003)	1,319 (95.997)	
Unemployed	634 (3.549)	99 (15.615)	535 (84.385)		19 (2.997)	615 (97.003)	
Others	6,694 (37.470)	1,342 (20.048)	5,352 (79.952)		262 (3.914)	6,432 (96.086)	
Living area, *n* (%)				**<0.001**			**<0.001**
Central urban area (where businesses gather or traffic is heavy)	6,300 (35.264)	1,559 (24.746)	4,741 (75.254)		377 (5.984)	5,923 (94.016)	
Other densely populated urban areas (residential areas)	6,117 (34.240)	1,228 (20.075)	4,889 (79.925)		242 (3.956)	5,875 (96.044)	
Suburban areas (rural areas)	4,120 (23.062)	878 (21.311)	3,242 (78.689)		225 (5.461)	3,895 (94.539)	
Others	1,328 (7.434)	272 (20.482)	1,056 (79.518)		47 (3.539)	1,281 (96.461)	
Living time, *n* (%)				**<0.001**			0.060
6 months−3 years	2,801 (15.679)	523 (18.672)	2,278 (81.328)		116 (4.141)	2,685 (95.859)	
>3–10 years	5,522 (30.910)	1,249 (22.619)	4,273 (77.381)		274 (4.962)	5,248 (95.038)	
>10 years	9,542 (53.412)	2,165 (22.689)	7,377 (77.311)		501 (5.250)	9,041 (94.750)	
City type, *n* (%)				**<0.001**			**<0.001**
National Sanitary Cities	11,782 (65.950)	2,754 (23.375)	9,028 (76.625)		654 (5.551)	11,128 (94.449)	
Non-National Sanitary Cities	6,083 (34.050)	1,183 (19.448)	4,900 (80.552)		237 (3.896)	5,846 (96.104)	
Regional economic situation, *n* (%)				**<0.001**			**<0.001**
Developed regions	4,886 (27.350)	1,283 (26.259)	3,603 (73.741)		345 (7.061)	4,541 (92.939)	
Moderately developed regions	7,912 (44.288)	1,673 (21.145)	6,239 (78.855)		355 (4.487)	7,557 (95.513)	
Underdeveloped regions	5,067 (28.363)	981 (19.361)	4,086 (80.639)		191 (3.769)	4,876 (96.231)	
Health literacy rate, %	16.600 (14.980, 21.300)	17.360 (14.980, 22.400)	16.600 (14.980, 21.185)	**<0.001**	16.600 (14.980, 22.400)	16.600 (14.980, 21.300)	**<0.001**
Average years of education, years	9.380 (8.800, 10.640)	9.600 (8.900, 10.640)	9.300 (8.800, 10.640)	**<0.001**	9.700 (9.000, 10.640)	9.380 (8.800, 10.640)	**<0.001**
Infant mortality rate, %	3.370 (2.840, 4.660)	3.400 (2.870, 4.660)	3.310 (2.840, 4.660)	**<0.001**	3.400 (2.870, 3.510)	3.370 (2.840, 4.660)	**0.014**

Bold font indicates statistically significant results (*P* < 0.05).

Multivariable logistic regression analysis at the 80% cut-off point identified associations with gender, educational level, age, occupation, living area, living time, regional economic situation, health literacy rate, average years of education, and infant mortality rate (*P* < 0.05 for the corresponding estimates in Table 2). City type was not significantly associated with satisfaction at the 80% threshold after adjustment (OR = 1.012, 95% CI: 0.903–1.135, *P* = 0.836). At the 90% threshold, residents in National Sanitary Cities had higher odds of satisfaction than residents in non-National Sanitary Cities (OR = 1.442, 95% CI: 1.157–1.796, *P* = 0.001), while infant mortality rate was not statistically significant (OR = 0.974, 95% CI: 0.905–1.048, *P* = 0.482; Table 2).

**Table 2:** Factors associated with residents' environmental sanitation satisfaction.

Variable	80% score as cut-off point			90% score as cut-off point		
	OR	95% CI	*P-*value	OR	95% CI	*P-*value
Gender						
Male	ref			ref		
Female	0.670	0.622–0.721	**<0.001**	0.660	0.574–0.759	**<0.001**
Educational level						
Junior high school and below	ref					
Technical secondary school/senior high school/technical school	1.221	1.070–1.395	**0.003**	1.464	1.100–1.949	**0.009**
Junior college/bachelor degrees and above	1.422	1.244–1.626	**<0.001**	2.029	1.530–2.691	**<0.001**
Age						
18–40	ref			ref		
41–60	1.131	1.041–1.228	**0.004**	1.127	0.965–1.315	0.131
≥60	1.243	1.044–1.479	**0.014**	1.500	1.062–2.118	**0.021**
Occupation						
Managers of government agencies, enterprises and institutions	ref			ref		
Professional technicians	0.663	0.598–0.734	**<0.001**	0.578	0.481–0.695	**<0.001**
Retired	0.863	0.716–1.040	0.121	0.534	0.364–0.783	**0.001**
Unemployed	0.563	0.446–0.713	**<0.001**	0.455	0.280–0.741	**0.002**
Others	0.728	0.657–0.806	**<0.001**	0.568	0.471–0.686	**<0.001**
Living area						
Central urban area (where businesses gather or traffic is heavy)	ref			ref		
Other densely populated urban areas (residential areas)	0.765	0.702–0.835	**<0.001**	0.663	0.560–0.785	**<0.001**
Suburban areas (rural areas)	0.874	0.792–0.965	**0.008**	0.983	0.822–1.175	0.849
Others	0.866	0.746–1.004	0.057	0.668	0.488–0.915	**0.012**
Living time						
6 months−3 years	ref			ref		
>3–10 years	1.266	1.127–1.422	**<0.001**	1.205	0.962–1.509	0.104
>10 years	1.172	1.048–1.311	**0.005**	1.164	0.937–1.444	0.169
City type						
Non-National Sanitary Cities	ref			ref		
National Sanitary Cities	1.012	0.903–1.135	0.836	1.442	1.157–1.796	**0.001**
Regional economic situation						
Developed regions	ref			ref		
Moderately developed regions	0.876	0.792–0.970	**0.011**	0.783	0.650–0.943	**0.010**
Underdeveloped regions	0.682	0.603–0.772	**<0.001**	0.637	0.502–0.809	**<0.001**
Health literacy rate	1.011	1.002–1.021	**0.020**	0.980	0.963–0.997	**0.023**
Average years of education	0.995	0.993–0.998	**0.001**	0.991	0.985–0.998	**0.006**
Infant mortality rate	1.096	1.057–1.136	**<0.001**	0.974	0.905–1.048	0.482
Constant	0.234		**<0.001**	0.089		**<0.001**

Bold font indicates statistically significant results (*P* < 0.05).

In the OLS robustness model using the continuous total satisfaction score, National Sanitary City status was not significantly associated with total satisfaction (*B* = −0.464, 95% CI: −1.263 to 0.335, *P* = 0.255). In the logistic models, National Sanitary City status was not significantly associated with reaching the 80% satisfaction threshold (OR = 1.012, 95% CI: 0.903–1.135, *P* = 0.836), but it was positively associated with reaching the 90% satisfaction threshold (OR = 1.442, 95% CI: 1.157–1.796, *P* = 0.001). The VIF values ranged from 1.06 to 2.99, indicating no severe multicollinearity among the model covariates. These results suggest that the association between National Sanitary City status and satisfaction is threshold-specific rather than uniform across all satisfaction definitions.

## 4 Discussion

The findings should be interpreted in a balanced way. Residents in National Sanitary Cities had a higher unadjusted proportion of satisfaction and were more likely to reach the stricter 90% satisfaction threshold after adjustment; however, National Sanitary City status was not significantly associated with the continuous total score or the 80% threshold. This pattern suggests that the policy designation may be more clearly related to achieving high-level satisfaction than to shifting overall satisfaction uniformly. At the same time, overall satisfaction levels remained low, highlighting the need for long-term policy research and targeted sanitation governance.

Residents' satisfaction with environmental sanitation is jointly shaped by a range of individual and contextual factors. Modern urban development faces pressing health, social, and environmental dilemmas, which highlights the necessity of city-level governance and systematic strategic planning to advance the Sustainable Development Goals proposed by the World Health Organization (26). The National Sanitary City program has helped policymakers better recognize the bidirectional link between environmental sanitation and public health, and promoted the incorporation of health perspectives into all urban policies (27). Cities awarded this title generally boast improved living and working infrastructure alongside higher resident health literacy. Given relatively steady external conditions and public expectations, resident satisfaction can act as a vital metric to evaluate local sanitation status and the practical effects of the sanitation city campaign (28), and it indirectly mirrors the long-term impacts of relevant urban sanitation policies. Nevertheless, our study data reveal that the mere designation of being a National Sanitary City cannot fully account for disparities in residents' sanitation satisfaction. While this initiative has deepened policymakers' understanding of how environmental conditions interact with population health, satisfaction gaps are simultaneously driven by other confounding variables. Therefore, demographic traits, socioeconomic status, residential location, length of residence, health literacy, average educational attainment and infant mortality rates must all be taken into consideration when interpreting satisfaction results.

Moreover, observations indicate that regardless of the 80 or 90% cut-off point for scoring, the proportion of residents satisfied with overall environmental sanitation remains low. Since satisfaction is a subjective metric shaped by individual perceptions, knowledge, and attitudes, it fails to accurately reflect the true condition of environmental sanitation or the efficacy of Sanitary City initiatives; instead, it highlights the gap between individual expectations and experiences. Studies have shown that the environment is closely related to human health (29), and the type of living environment is the basic spatial carrier for residents' daily lives, encompassing various aspects such as facilities, transportation, and interactions between people and the environment. Previous research has indicated that the living environment can have direct or indirect effects on people's physical and mental health, and improvements in urban environments can play a positive role in promoting public health. Residents living in communities with poor environments are more likely to experience negative health impacts (30). Therefore, enhancing the urban environmental sanitation is of great significance. However, it is worth noting that residents' subjective perceptions of the urban environment do not completely align with the results measured by objective indicators (31), and this inconsistency is quite common. The reason may be that objective and perceptual measurements may capture different aspects of the environment, and objective indicators are not an effective way to reflect people's perceptual assessments (32). Thus, subjective perceptual indicators can better reflect the problems existing in the details of urban environmental sanitation and are more instructive for the formulation and adjustment of future policies. Although the selected National Sanitary Cities have performed well in objective indicator evaluations, there is still a gap between these evaluations and residents' expectations.

This pattern suggests that policy designation and objective sanitation improvement do not automatically guarantee strong perceived gains in everyday life. A people-centered interpretation of National Sanitary City construction places residents' experiences within the policy evaluation process. Urban sanitation governance addresses health, social, and environmental challenges through planning, management, and intersectoral action. Residents' satisfaction can complement formal standards by indicating whether sanitation infrastructure, public-place hygiene, and management efforts are visible and meaningful to people in their daily environments.

Residents with higher education levels, such as managers of government agencies, enterprises, and institutions, especially those in central urban areas of developed regions, typically reported greater satisfaction with environmental sanitation. This to some extent reflects that residents with better economic levels and good education have higher satisfaction. The reasons for this outcome may be that these residents possess more knowledge about environmental sanitation and have better economic conditions to access a wider range of environments, with less restriction from the regional environmental scope. This allows residents to have a stronger sense of gain, which has a positive effect on enhancing their satisfaction. This higher satisfaction partly stems from their better socioeconomic status and residence in more developed city areas, where their expectations more closely align with their actual experiences. It is worth noting that health literacy rate was positively associated with satisfaction at the 80% threshold but negatively associated at the 90% threshold, while the estimate for infant mortality rate changed direction and lost statistical significance at the 90% threshold. One possible interpretation is that basic satisfaction and very high satisfaction reflect different expectation levels or regional contexts.

Previous research on National Sanitary Cities primarily evaluated the effects of the policy (33), including environmental sanitation (34, 35), vector control (36), and infectious disease prevention, but seldom addressed environmental sanitation from the residents' subjective perspectives. This study, from the standpoint of primary stakeholders in establishing National Sanitary Cities, investigates the principal factors influencing residents' satisfaction with environmental sanitation. It seeks to use the firsthand experiences of those affected by environmental conditions to identify issues in the creation process of National Sanitary Cities, thereby guiding future initiatives.

This study has several limitations. Although it covered a broad geographic area with a substantial sample size, the central intercept survey method may constrain participant representativeness. In addition, data on some variables including waste management service quality, drainage water quality and population density were unavailable due to limited data accessibility and thus excluded from analysis. The available measures also emphasize residents' subjective satisfaction and a limited set of area-level indicators, while more detailed objective sanitation measures were unavailable. The sampling structure is nested across administrative units; however, the city-level analytic sample included a limited number of higher-level units. In this context, multilevel random-effect estimates or clustered robust standard errors at the city or provincial level may be unstable. The present analysis was therefore designed to identify adjusted associations under threshold-defined satisfaction outcomes rather than to estimate variance components or cross-level effects. Future studies with more higher-level units and richer contextual measurements should further examine clustering, cross-level effects, and the stability of regional associations.

## 5 Conclusion

Residing in a National Sanitary City was positively correlated with residents reaching the stricter 90% satisfaction threshold, yet overall satisfaction remained limited. Policy improvement should therefore combine formal sanitation standards with routine resident feedback, focus on the sanitation details most visible in daily life, and use satisfaction monitoring to identify groups and places where perceived gains lag behind administrative progress. This people-centered approach can support more targeted and sustainable National Sanitary City governance.

## Data Availability

The raw data supporting the conclusions of this article will be made available by the authors, without undue reservation.

## References

[1] Khalil L Abbas S Hussain K Zaman K Iswan Salamun H . Sanitation, water, energy use, and traffic volume affect environmental quality: go-for-green developmental policies. PLoS ONE. (2022) 17:e0271017. doi: 10.1371/journal.pone.0271017 36026488 PMC9417191

[2] Sinharoy SS Pittluck R Clasen T. Review of drivers and barriers of water and sanitation policies for urban informal settlements in low-income and middle-income countries. Util Policy. (2019) 60:100957. doi: 10.1016/j.jup.2019.100957 32214692 PMC7067261

[3] Taşkaya S. Environmental quality and well-being level in Turkey. Environ Sci Pollut Res Int. (2018) 25:27935–44. doi: 10.1007/s11356-018-2806-4 30058040

[4] Sierra-Vargas MP Teran LM. Air pollution: impact and prevention. Respirology. (2012) 17:1031–8. doi: 10.1111/j.1440-1843.2012.02213.x 22726103 PMC3532603

[5] Wang Y Ding X Chen Y Zeng W Zhao Y. Pollution source identification and abatement for water quality sections in Huangshui River basin, China. J Environ Manage. (2023) 344:118326. doi: 10.1016/j.jenvman.2023.118326 37329584

[6] Thompson R Smith RB Bou Karim Y Shen C Drummond K Teng C . Noise pollution and human cognition: an updated systematic review and meta-analysis of recent evidence. Environ Int. (2022) 158:106905. 34649047 10.1016/j.envint.2021.106905

[7] Anand N Palani SG. A comprehensive investigation of toxicity and pollution potential of municipal solid waste landfill leachate. Sci Total Environ. (2022) 838(Pt 1):155891. doi: 10.1016/j.scitotenv.2022.155891 35568169

[8] Cardozo RN. An experimental study of customer effort, expectation, and satisfaction. J Mark Res. (1965) 2:244–9. doi: 10.1177/002224376500200303

[9] Veitch J Rodwell L Abbott G Carver A Flowers E Crawford D. Are park availability and satisfaction with neighbourhood parks associated with physical activity and time spent outdoors? BMC Public Health. (2021) 21:306. doi: 10.1186/s12889-021-10339-1 33549088 PMC7866776

[10] Parra DC Van Zandt A Wang P Goodman M Abhishek J Haire-Joshu D . Evaluating park use and satisfaction: the Case of Trojan Park in St. Louis Missouri. Int J Environ Res Public Health. (2019) 16:2798. doi: 10.3390/ijerph16152798 31390742 PMC6696297

[11] Shafer CS Lee BK Turner S. A tale of three greenway trails: user perceptions related to quality of life. Landsc Urban Plan. (2000) 49:163–78. doi: 10.1016/S0169-2046(00)00057-8

[12] Park JH Choi JM. The effect of residential environment satisfaction on depression in the elderly: focusing on the mediating effect of stress. Front Public Health. (2022) 10:1038516. doi: 10.3389/fpubh.2022.1038516 36268005 PMC9577004

[13] Chen L Zhang J You Y. Air pollution, environmental perceptions, and citizen satisfaction: a mediation analysis. Environ Res. (2020) 184:109287. doi: 10.1016/j.envres.2020.109287 32155488

[14] Orru K Orru H Maasikmets M Hendrikson R Ainsaar M. Well-being and environmental quality: does pollution affect life satisfaction? Qual Life Res. (2016) 25:699–705. doi: 10.1007/s11136-015-1104-6 26289023

[15] Yao L Li X Zheng R Zhang Y. The impact of air pollution perception on urban settlement intentions of young talent in China. Int J Environ Res Public Health. (2022) 19:1080. doi: 10.3390/ijerph19031080 35162103 PMC8834384

[16] Wang Y Zhu Y Yu M. Evaluation and determinants of satisfaction with rural livability in China's less-developed eastern areas: a case study of Xianju County in Zhejiang Province. Ecol. Indic. (2019) 104:711–22. doi: 10.1016/j.ecolind.2019.05.054

[17] Zhao X Sun H Chen B Xia X Li P. China's rural human settlements: qualitative evaluation, quantitative analysis and policy implications. Ecol. Indic. (2019) 105:398–405. doi: 10.1016/j.ecolind.2018.01.006

[18] Wang P Qin X Li Y. Satisfaction evaluation of rural human settlements in northwest China: method and application. Land. (2021) 10:813. doi: 10.3390/land10080813

[19] Zheng WJ Qi X Yao HY Liu JJ Yu SC. Analysis on the current situation and influencing factors of residents' satisfaction with the built environment of China's Hygienic City Initiative. Zhonghua Yu Fang Yi Xue Za Zhi. (2023) 57:1820–6. (in Chinese). doi: 10.3760/cma.j.cn112150-20221113-01104 38008572

[20] National Health Commission of the People's Republic of China. National Sanitary Cities and Counties List (as of December 2020). National Health Commission of the People's Republic of China. Available online at: https://www.nhc.gov.cn/wjw/gjwscz/202107/b3cf9f3d97e6477fb1f2ab85d80ceaca.shtml. (in Chinese). (Accessed August 15, 2025).

[21] Zheng WJ Qi X Yao HY Liu JJ Yu SC Zhang T. Influence of built environment in hygienic city in China on self-rated health of residents. Biomed Environ Sci. (2022) 35:1126–32. doi: 10.3967/bes2022.142 36597292

[22] National Health Commission of the People's Republic of China. Notice on the Review Management Measures for National Sanitary Towns and the Standards for National Sanitary Cities and National Sanitary Counties (2021). (In Chinese).

[23] World Health Organization (1991). Creating Healthy Cities. Geneva: World Health Organization (Accessed May 21, 2026).

[24] Dong JX Wang QQ Yao HY Sun JF Qi X Jiang DX . Development, reliability and validity evaluation of residents' satisfaction questionnaire on environmental sanitation of National Hygienic City. Chin J Health Educ. (2022). 38:776–81. doi: 10.16168/j.cnki.issn.1002-9982.2022.09.002

[25] Dong JX. Development and empirical study of a tool for assessing the satisfaction of residents in environmental sanitation. Chin Center Dis Control Prev. (2024).

[26] Lowe M Adlakha D Sallis JF Salvo D Cerin E Moudon AV . City planning policies to support health and sustainability: an international comparison of policy indicators for 25 cities. Lancet Glob Health. (2022) 10:e882–94. doi: 10.1016/S2214-109X(22)00069-9 35561723 PMC9906636

[27] Ruan SM Yue DH Cheng G Zhu WM Meng QY. Influence of China healthy cities movement on health of local residents. J Environ Health. (2015). 32:142–6. (In Chinese). doi: 10.16241/j.cnki.1001-5914.2015.02.017

[28] Mao TJ Hu W Yang F. Analysis of the role of public satisfaction survey in the long-term management of National Sanitary Cities. Zhejiang Prev Med. (2015) 27:529–31. (in Chinese). doi: 10.19485/j.cnki.issn1007-0931.2015.05.033

[29] Laddu D Paluch AE LaMonte MJ. The role of the built environment in promoting movement and physical activity across the lifespan: implications for public health. Prog Cardiovasc Dis. (2021) 64:33–40. doi: 10.1016/j.pcad.2020.12.009 33428966

[30] Lu S Liu Y Guo Y Ho HC Song Y Cheng W . Neighborhood built environment and late-life depression: a multilevel path analysis in a Chinese society. J Gerontol B Psychol Sci Soc Sci. (2021) 76:2143–54. doi: 10.1093/geronb/gbab037 33674824

[31] Koohsari MJ Badland H Sugiyama T Mavoa S Christian H Giles-Corti B. Mismatch between perceived and objectively measured land use mix and street connectivity: associations with neighborhood walking. J Urban Health. (2015) 92:242–52. doi: 10.1007/s11524-014-9928-x 25539783 PMC4411311

[32] Smith G Gidlow C Davey R Foster C. What is my walking neighbourhood? A pilot study of English adults' definitions of their local walking neighbourhoods. Int J Behav Nutr Phys Act. (2010) 7:34. doi: 10.1186/1479-5868-7-34 20459636 PMC2873577

[33] Zhang DX Peng HY Wu W. Exploration of the role of health impact assessment in patriotic health work in the new era. Jiangsu Prev Med. (2023) 34:366–8. (in Chinese). doi: 10.13668/j.issn.1006-9070.2023.03.037

[34] Yu J Feng L Zheng CL Tian ZY Zhang Y Huang GF. Analysis on the results of check unannounced to the national health cities (countries) in Yunnan Province in 2021. J Med Pest Control. (2024) 40:683–7. (in Chinese).

[35] Cheng SX Wang SK Jiang JX Liu LF Lv J. Surveillance and analysis of drinking water quality before and after creating National Health City. Chin J Health Lab Tec. (2019) 29:2003–5. (in Chinese).

[36] Zhang XT Wu JH. An analysis of influence of National Sanitary City construction on vector control in Baiyin, Gansu province, China. Chin J Vector Biol Control. (2022) 33:715–21. (in Chinese).

